# Personal, social and environmental correlates of healthy weight status amongst mothers from socioeconomically disadvantaged neighborhoods: findings from the READI study

**DOI:** 10.1186/1479-5868-7-23

**Published:** 2010-03-23

**Authors:** Abbie MacFarlane, Gavin Abbott, David Crawford, Kylie Ball

**Affiliations:** 1 Centre for Physical Activity and Nutrition Research, School of Exercise and Nutrition Sciences, Deakin University, Australia

## Abstract

**Background:**

Socioeconomically disadvantaged mothers are at high risk of obesity, yet the aetiology of obesity in this group remains poorly understood. The aim of this study was to examine the perceived personal, social and physical environmental factors associated with resilience to obesity among mothers from socioeconomically disadvantaged neighbourhoods.

**Methods:**

Survey data were provided by a cohort of 1840 women aged 18-46 years with dependent children (aged 0-18 years) from 40 urban and 40 rural socioeconomically disadvantaged neighbourhoods across Victoria, Australia. Mothers responded to a number of questions relating to personal, social and environmental influences on their physical activity and eating habits. Mothers' weight status was classified as healthy weight (BMI: 18.5-24.99), overweight (BMI: 25-29.99) or obese (BMI: 30+).

**Results:**

Mothers' weight status was bivariably associated with factors from all three domains (personal, social and physical environmental). In a multivariable model, mothers' perceived ability to make time for healthy eating (OR = 1.34) and physical activity (OR = 1.11) despite family commitments, and the frequency with which families ate healthy low-fat foods with mothers (OR = 1.28) remained significantly positively associated with healthy weight status. The frequency with which families encouraged eating healthy low-fat foods remained negatively associated (OR = 0.81) with weight status; ie greater encouragement was associated with less healthy weight status.

**Conclusions:**

Drawing on the characteristics of mothers resilient to obesity might assist in developing intervention strategies to help other mothers in socioeconomically disadvantaged neighbourhoods to manage their weight. Such strategies might focus on planning for and prioritising time for healthy eating and physical activity behaviours, and including family members in and encouraging family mealtimes.

## Background

The prevalence of overweight and obesity is increasing worldwide [1-4], with certain population subgroups at particular risk. Women of child-bearing age (around 18-45 years), particularly those with children, are not only at high risk of weight gain due to pregnancy [5-8], but experience a high risk of physical inactivity [9], unhealthy eating patterns [10], and the greatest barriers to adopting healthy lifestyle change [11]. Mothers who are socioeconomically disadvantaged, or living in socioeconomically disadvantaged neighbourhoods, may face even greater risk of obesity, since previous studies have demonstrated that neighbourhood deprivation is associated with obesity risk, independently of individual-level socioeconomic position [12-14] and that physical inactivity and poor diet are disproportionately experienced by these groups [15-19].

Despite the high risk of obesity amongst mothers from socioeconomically disadvantaged neighbourhoods, the aetiology of obesity in this group remains poorly understood. Although eating and physical activity are believed to be key behaviours implicated in the aetiology of weight gain [9-11], a finding consistent with our recent report on the demographic and behavioural correlates of weight status amongst socioeconomically disadvantaged mothers [20], the personal, social and physical-environmental influences on these behaviours and on weight during this life-stage are not well-understood. Better knowledge of the modifiable correlates of obesity amongst this target group is critical in order to plan and implement effective obesity prevention initiatives.

A range of potential factors might influence the weight status of mothers from socioeconomically disadvantaged neighbourhoods. Several recent qualitative studies have identified personal factors, such as neglect of self-care over commitment to looking after other family members, as impacting on physical activity or healthy eating and potentially playing an important role in the development of obesity in low-income mothers [10,21]. Similarly, other evidence demonstrates that mothers often report numerous barriers to health behaviours associated with their mothering role and family commitments [11,22].

Additionally, social support from partners and family has been found to positively predict healthy eating, physical activity and weight loss among low-income mothers [21,23,24]. One aspect of the physical environment that has been suggested in qualitative research to influence the eating behaviour and weight of low-income mothers is the availability of foods in the home [21], with mothers reporting that they ate any foods available (e.g., sweetened beverages, mini-donuts) at home throughout the day.

Given the complex array of potential personal, social and environmental influences on obesity, researchers have called for theoretical approaches to studying obesity and obesity risk behaviours [25,26]. Many recent theory-based studies investigating possible predictors of obesity have been grounded in social ecological theory (SET) [27]. According to SET, health outcomes such as obesity are influenced by not only intrapersonal factors such as attitudes or behaviours, but also by a broad array of social and physical environmental conditions. Social ecological theory emphasises the importance of these multiple domains and their dynamic interactions in determining health and illness. However, to our knowledge only one previous study has applied SET to the study of obesity among socioeconomically disadvantaged mothers [21] and this qualitative study was focused on identifying the predictors of obesity or unhealthy weight gain. To understand the necessary elements of effective prevention, however, it is also useful to identify characteristics of mothers who manage, despite the odds, to avoid unhealthy weight gain. To our knowledge, no research to date has quantitatively tested whether constructs of SET explain variability in body weight and 'resilience' to obesity amongst mothers from socioeconomically disadvantaged neighbourhoods.

The purpose of this study was to identify the perceived personal, social environmental and physical environmental factors associated with healthy weight status among a large sample of mothers from socioeconomically disadvantaged neighbourhoods. It was hypothesized that among these mothers, those who are in the healthy weight range, despite their increased odds of being overweight, would be characterised by the following:

(1) Personal factors: Greater commitment to prioritising own self-care related to healthy eating and physical activity in balance with family commitments (e.g. more likely to make time for healthy eating and physical activity despite family commitments);

(2) Social environmental factors: higher perceived social support from family for healthy eating and physical activity behaviours; higher frequency of family meals; lower perceived likelihood of being influenced by children's food preferences;

(3) Physical environmental factors: greater home availability of healthy foods (e.g. fruit and veg) and lower home availability of unhealthy foods (e.g. energy-dense snacks and drinks).

## Methods

### Sample

This study used baseline data derived from the Resilience for Eating and Activity Despite Inequality (READI) study, a longitudinal cohort study examining resilience to obesity among women and children from socioeconomically disadvantaged neighbourhoods. Study methods are described in detail elsewhere [20] and summarized here. The READI study involves a cohort of women aged 18-46 years who were randomly selected using the electoral roll (voting is compulsory in Australia) from 40 rural and 40 urban areas of Victoria identified as socioeconomically disadvantaged using the Socioeconomic Index for Areas (SEIFA) developed by the Australian Bureau of Statistics from census data [28]. All Victorian suburbs were split into two groups, urban and rural, and ranked according to disadvantage. For each group, suburbs in the lowest disadvantage tertile, that had populations of 1,200 people or more (to optimise the chance or an adequately sized sampling pool) and that were within 200 km of Melbourne, formed the READI study's suburb selection pool. Subsequently, 40 urban and 40 rural suburbs were randomly selected as the final sampling suburbs, and 150 women within the target age range from each suburb were randomly selected from the electoral roll to participate. In three suburbs where there were fewer than 150 eligible women, all eligible women were invited to take part, resulting in a total sampling pool of 11940 women.

Between August 2007 and January 2008, a total of 4934 women completed a survey which assessed a broad range of factors that might influence women's obesity risk. Excluding from the denominator those whose surveys were returned to sender (address unknown; n = 861) or who were otherwise ineligible (e.g., were deceased, did not meet study criteria for sex; n = 17), this represented a response rate of 45%. Of these women, 571 of the respondents no longer resided within one of the 80 study suburbs, nine were excluded because they were not within the desired age range (18-46 years), three surveys were completed by women other than those to whom they were addressed, and two women subsequently withdrew from the study. Data from these women were excluded from analyses, leaving data from 4349 women. Of this group, 2648 (61%) reported that they had at least one dependent child (aged 0-18 years). Data from mothers who were pregnant at the time of the survey (n = 129), were menopausal (n = 89), did not provide height and weight data (n = 159), were underweight (n = 66) or had incomplete data on relevant sociodemographic or predictor variables (n = 484) were also excluded from the analyses. Taking into account that some women met two or more of these exclusion conditions, the total number of mothers whose data were excluded was 808, leaving 1840 mothers for whom data were analysed in this study. Comparison of these 1840 mothers with those who were excluded showed no significant differences on the key study outcome (weight status).

### Procedure

Initially, 11940 women were mailed a survey package. To maximise response rates, a reminder protocol [29] was employed whereby letters were sent to non-responders ten days after the initial survey package was mailed, followed by a second reminder letter including another copy of the survey a further ten days later.

### Measures

The READI survey included questions about height and weight, sociodemographic characteristics and a range of personal, social environmental and physical environmental factors hypothesized to be associated with women's eating, physical activity and sedentary behaviours. Survey development was guided by a theoretical framework (SET) for understanding factors influencing eating, physical activity and sedentary behaviour, a review of the literature for existing instruments, and pilot-testing of the survey with a convenience sample of 32 women aged 18-46 years. Consistent with SET, we selected predictor variables that captured not only intrapersonal factors, but also aspects of the social and physical environments in which women live that might plausibly influence obesity risk. The survey items used in the present study, which were selected based on these factors and their high relevance to motherhood, are presented below.

#### Outcome measure

Mothers' weight status, indicated by their body mass index (BMI) category, was used as the outcome variable in the analyses. BMI (weight [kg]/height [m^2^]) was calculated from self-reported height and weight, which have been reported to be correlated with measured height and weight (*r *> 0.9) [30] and generally considered an appropriate method for epidemiological studies where objective measurement is less feasible. Mothers were classified as healthy weight (BMI = 18.50-24.99), overweight (BMI = 25.00-29.99) or obese (BMI ≥ 30.00), according to BMI cut-points developed by the World Health Organization [1]. Underweight mothers (BMI < 18.50; n = 66) were excluded from analyses since this study aimed to compare characteristics of healthy weight (or obesity resilient) and overweight or obese (non-obesity resilient) mothers.

#### Predictor measures

Personal correlates were examined through a set of six questions that addressed the priority mothers gave to self-care related to healthy eating and physical activity. Social environmental correlates were assessed by ten questions that addressed family support for healthy eating and physical activity, frequency of family meals, and children's food preferences. Physical environmental correlates were based on four questions assessing the availability of healthy and unhealthy foods in the home. Table 1 presents the specific survey items and their response options for all predictor measures, and Cronbach's alphas (internal consistency) for two compound predictor variables, availability of energy-dense snacks and availability of energy-dense drinks.

**Table 1:** Description of survey items for potential personal, social-environmental and physical-environmental correlates of weight status

Factors	Description of survey items
**PERSONAL**	
Self-care related to healthy eating	1. "I often feel guilty preparing healthy food when my family would prefer to eat other kinds of foods." 5 responses ranging from "strongly disagree" to "strongly agree." Mean: 2.0 ± 1.0; range: 1-5. 2. "My family's food preferences usually take priority over my own food preferences." 5 responses ranging from "strongly disagree" to "strongly agree." Mean: 2.8 ± 1.2; range: 1-5. 3. "I make time to eat healthy foods even when I am busy looking after my family." 5 responses ranging from "strongly disagree" to "strongly agree." Mean: 3.6 ± 1.0; range: 1-5.
	
Self-care related to physical activity	1. "I often feel guilty doing physical activity when I have family commitments." 5 responses ranging from "strongly disagree" to "strongly agree." Mean: 3.2 ± 1.2; range: 1-5. 2. "My family commitments usually take priority over my physical activity." 5 responses ranging from "strongly disagree" to "strongly agree." Mean: 3.9 ± 1.1; range: 1-5. 3. "I make time for physical activity even when I am busy with family commitments." 5 responses ranging from "strongly disagree" to "strongly agree." Mean: 2.8 ± 1.2; range: 1-5.
	
**SOCIAL ENVIRONMENTAL**	
Family support for healthy eating	1. "During the past year, how often did members of your family eat healthy low-fat foods with you?" 5 responses ranging from "never" to "very often." Mean: 3.9 ± 1.1; range: 1-5. 2. "During the past year, how often did members of your family encourage you to eat healthy low-fat foods?" 5 responses ranging from "never" to "very often." Mean: 3.1 ± 1.3; range: 1-5. 3. "During the past year, how often did members of your family discourage you from eating unhealthy foods?" 5 responses ranging from "never" to "very often." Mean: 2.6 ± 1.3; range: 1-5.
	
Family or friend support for physical activity	1. "During past year, how often did family members do physical activity with you?" 5 responses ranging from "never" to "very often." Mean: 2.9 ± 1.3; range: 1-5. 2. "During past year, how often did family members encourage you to be physically active?" 5 responses ranging from "never" to "very often." Mean: 3.1 ± 1.3; range: 1-5. 3. "During past year, how often did family members discourage you from sitting around too much?" 5 responses ranging from "never" to "very often." Mean: 2.0 ± 1.2; range: 1-5. 4. "If you wanted to do physical activity without your children, do you have access to childcare either at a childcare centre, a partner/family member or a friend?" 3 responses: "yes", "no" and "not applicable" (i.e., I do not do physical activity at all/I would not do physical activity without my children). Distribution: "yes" (69.9%), "no" (20.1%), "not applicable" (10.0%).
	
Frequency of family meals	1. "How often do you usually eat dinner together with your family?" 6 response options were collapsed into three categories: "≤ 3 times/week" (rarely/never, less than once a week, about 1-3 times a week), "about 4-6 times/week" (about 4-6 times a week), and "daily" (everyday). Distribution: "≤ 3 times/week" (14.3%), "about 4-6 times/week" (29.9%), and "daily" (55.8%).
	
Children's influence	1. "I like to eat fruit because my children like to eat them." 7 response options were collapsed into 2 categories: "agree" (strongly agree, agree), "other" (neither agree nor disagree, disagree, strongly disagree, I don't eat fruit, not applicable). Distribution: "agree" (79.6%), "other" (20.4%). 2. "I like to eat vegetables because my children like to eat them." 7 response options were collapsed into 2 categories: "agree" (strongly agree, agree), "other" (neither agree nor disagree, disagree, strongly disagree, I don't eat fruit, not applicable). Distribution: "agree" (65.1%), "other" (34.9%).
	
**PHYSICAL ENVIRONMENTAL**	
Home food availability	1. "About how often are fruits available in your home?" 4 response options were collapsed into 2 categories: "always" (always), "other" (usually, sometimes, never). Distribution: "always" (80.5%), "other" (19.5%). 2. "About how often are vegetables available in your home?" 4 response options were collapsed into 2 categories: "always" (always), "other" (usually, sometimes, never). Distribution: "always" (88.7%), "other" (11.3%). 3. "About how often are these foods available in your home?... (a) cakes/doughnuts/biscuits, (b) potato chips or other salty snack foods, (c) chocolate or other lollies." 3 item scale, *How often are energy-dense snacks available in home?*, with response options ranging from "never" to "always". Cronbach's alpha = 0.68. Mean: 7.3 ± 1.7; range: 3-12. 4. "About how often are these foods available in your home?... (a) fruit juice, (b) soft drink, (c) sports drinks or energy drinks." 3 item scale, *How often are energy-dense snacks available in home?*, with response options ranging from "never" to "always". Cronbach's alpha = 0.44. Mean: 6.8 ± 1.8; range: 3-12.

#### Sociodemographic characteristics

The following sociodemographic characteristics were controlled for in multivariable regression analyses: age, country of birth ('Australia' or 'other', since the numbers in any single 'other' category were too small to analyse separately), and education level ('low' - up to Year 10 or equivalent; 'medium' - Year 12 and/or a technical or trade certificate/apprenticeship; or 'high' - university/higher university degree).

### Data analysis

Separate bivariable analyses were conducted to examine associations between mothers' weight status and 'obesity-resilience' (healthy BMI category), and each personal, social environmental and physical environmental predictor variable using single predictor ordinal regression. Ordinal regression analysis was selected because of the ordered and categorical nature of the dependent variable [31] (i.e., BMI category was ordered from obese (least healthy weight status) to overweight to healthy weight). Any predictor variables that were found to be significantly associated with weight status (p < 0.05) were entered into a multivariable ordinal regression model, adjusting for potential confounding factors of age, country of birth, and education level. To adjust for the clustering effects resulting from sampling from neighbourhoods, standard errors were computed using the Taylor-series approximation. All analyses were conducted using STATA 9.2 (Statacorp, Texas, USA).

### Statement of Ethics

All applicable institutional and governmental regulations concerning the ethical use of human volunteers were followed during this research. The READI study was approved by the Deakin University Human Research Ethics Committee, the Victorian Department of Education and the Catholic Education Office.

## Results

The mean age of mothers was 37.6 years (s.d. = 6.4). Most of the mothers were born in Australia (89.5%) and had a medium level of education (70.3%). About half of the mothers were in the healthy BMI range, while 28% and 24% were overweight and obese respectively.

Table 2 shows the bivariable associations between personal, social and physical environmental factors and weight status of mothers. Most of the personal variables and fewer than half of the social environmental and physical environmental factors were significantly associated with mothers' weight status. Healthy weight status was associated with greater commitment to self-care related to healthy eating. For example, mothers' disagreement with statements that they felt guilty for preparing healthy foods when their family would prefer other foods, and that their family's food preferences took priority over their own, were associated with healthy weight status. In addition, healthy weight status was associated with mothers' agreement that they made time to eat healthy foods despite family commitments. Healthy weight status was also associated with greater commitment to self-care related to physical activity, although for only one of the indicators assessed. Healthy weight status was associated with mothers' agreement that they were able to make time for physical activity despite family commitments.

**Table 2:** Means (SDs) or frequencies of personal, social environmental and physical environmental factors by weight status

Factors	BMI category						P^a^
		
	Healthy weight		Overweight		Obese		
**PERSONAL**							
*Self-care related to healthy eating *(mean, SD; range 1[strongly disagree] -5 [strongly agree])							
Feel guilty for preparing healthy foods when family prefers to eat other foods	1.91	(0.94)	2.01	(0.92)	2.17	(1.00)	**<0.0005**
Family's food preferences take priority over own food preferences	2.72	(1.20)	2.86	(1.17)	3.08	(1.19)	**<0.0005**
Make time to eat healthy foods even when busy looking after family	3.75	(1.00)	3.55	(1.01)	3.19	(1.01)	**<0.0005**
*Self-care related to PA *(mean, SD; range 1 [strongly disagree] -5 [strongly agree])							
Feel guilty doing PA when I have family commitments	3.14	(1.17)	3.20	(1.20)	3.16	(1.23)	0.650
Family commitments take priority over PA	3.89	(1.07)	3.96	(1.05)	4.00	(1.08)	0.057
Make time for PA even when busy with family commitments	2.88	(1.19)	2.80	(1.14)	2.56	(1.13)	**<0.0005**
**SOCIAL-ENVIRONMENTAL**							
*Family support for healthy eating *(mean, SD; range 1 [never] -5 [very often])							
How often does family eat healthy low-fat foods with you?	4.00	(1.03)	3.85	(1.05)	3.58	(1.11)	**<0.0005**
How often does family encourage you to eat healthy low-fat foods?	2.97	(1.34)	3.12	(1.30)	3.15	(1.27)	**0.017**
How often does family discourage you from eating unhealthy foods?	2.45	(1.25)	2.63	(1.25)	2.66	(1.26)	**0.001**
*Family/friend/environment support for PA *(mean, SD; range 1 [never] -5 [very often])							
How often does family do PA with you?	2.94	(1.26)	3.07	(1.27)	2.78	(1.30)	0.209
How often does family encourage you to do PA?	3.04	(1.31)	3.20	(1.31)	3.11	(1.28)	0.131
How often does family discourage you from sitting around?	1.89	(1.14)	2.00	(1.16)	2.08	(1.20)	**0.001**
Do you have access to childcare (centre/partner/family/friend) if you wanted to do PA? (%, n)							
Yes	71.5	(641)	71.9	(364)	64.5	(282)	0.110
No	19.4	(174)	18.2	(92)	23.6	(103)	
N/A	9.1	(82)	9.9	(50)	11.9	(52)	
*Frequency of family meals *(%, n)							
How often do you eat dinner with family?							
≤ 3 times/week	14.4	(129)	12.6	(64)	16.0	(70)	0.851
About 4-6 times/week	29.5	(265)	30.4	(154)	30.0	(131)	
Daily	56.1	(503)	56.9	(288)	54.0	(236)	
*Influence of children's food preferences *(%, n)							
I eat fruit because my children do							
Agree/strongly agree	78.5	(704)	82.6	(418)	78.5	(343)	0.607
Other	21.5	(193)	17.4	(88)	21.5	(94)	
I eat vegetables because my children do							
Agree/strongly agree	64.4	(578)	67.2	(340)	63.8	(279)	0.898
Other	35.6	(319)	32.8	(166)	36.2	(158)	
**PHYSICAL-ENVIRONMENTAL**							
*Home food availability *(%, n)							
How often is fruit available in home							
Always	81.8	(734)	82.2	(416)	75.7	(331)	0.057
Other	18.2	(163)	17.8	(90)	24.3	(106)	
How often are vegetables available in home (%, n)							
Always	88.3	(792)	90.3	(457)	87.6	(383)	0.966
Other	11.7	(105)	9.7	(49)	12.4	(54)	
How often are energy-dense snacks available in home? (mean, SD; range 3-12)	7.33	(1.72)	7.29	(1.65)	7.39	(1.65)	0.650
How often are energy-dense drinks available in home? (mean, SD; range 3-12)	6.67	(1.74)	6.74	(1.79)	7.06	(1.80)	**<0.0005**

^a ^P-values were calculated using ordinal logistic regression; P-values < 0.05 are bolded.

Abbreviations: PA = physical activity; N/A = not applicable

Of the ten social environmental variables, four were significantly bivariably associated with mothers' weight status. Healthy weight status was associated with mothers' families more frequently eating healthy low-fat foods with them, and less frequently encouraging them to eat healthy low-fat foods, discouraging them from eating unhealthy foods, and discouraging them from sitting around. Of the four physical environmental factors assessed, only availability of energy-dense drinks in the home was associated bivariably with weight status, with lower availability associated with healthy weight.

Table 3 shows the results of the multivariable ordinal regression model predicting the likelihood of mothers being of healthy weight status, after adjusting for potential confounding factors of age, country of birth, and education level and all other variables significantly bivariably associated with weight status. Four correlates remained significant in the multivariable model. Making time to eat healthy foods even when busy looking after family (OR = 1.34, CI = 1.21-1.47, p < 0.0005); and making time for physical activity even when busy with family commitments (OR = 1.11, CI = 1.02-1.20, p = 0.016), were significantly positively associated with mothers' healthy weight status. Similarly, greater frequency of families eating healthy low-fat foods with women (OR = 1.28, CI = 1.16-1.41, p < 0.0005) was positively associated with healthy weight status. Frequency of families encouraging women to eat healthy low-fat foods (social environmental) was also associated with weight status (OR = 0.81, CI = 0.73-0.89, p < 0.0005), such that less frequent family encouragement was associated with healthier weight status.

**Table 3:** Multivariate associations between personal, social environmental and physical environmental correlates and weight status

Factors	**Adjusted OR**^a^	Adjusted 95% CI	P^b^
**PERSONAL**			
*Self-care related to healthy eating*			
Feel guilty for preparing healthy foods when family prefers to eat other foods	0.94	(0.84, 1.05)	0.258
Family's food preferences take priority over own food preferences	0.96	(0.87, 1.06)	0.418
Make time to eat healthy foods even when busy looking after family	1.34	(1.21, 1.47)	**<0.0005**
*Self-care related to PA*			
Make time for PA even when busy with family commitments	1.11	(1.02, 1.20)	**0.016**
**SOCIAL-ENVIRONMENTAL**			
*Family support for healthy eating*			
How often does family eat healthy low-fat foods with you?	1.28	(1.16, 1.41)	**<0.0005**
How often does family encourage you to eat healthy low-fat foods?	0.81	(0.73, 0.89)	**<0.0005**
How often does family discourage you from eating unhealthy foods?	0.98	(0.89, 1.07)	0.624
*Family/friend/environment support for PA*			
How often does family discourage you from sitting around?	0.98	(0.91, 1.06)	0.652
**PHYSICAL-ENVIRONMENTAL**			
*Home food availability*			
How often are energy-dense drinks available in home?	0.96	(0.92, 1.01)	0.087

Adjusted for country of birth, age, maternal education (co-variates), clustering of mothers by suburb, and all other predictor variables listed.

^a ^Odds ratios (ORs) > 1 indicated that higher scores on the measure (e.g. greater agreement with self care statements, frequency of family support, or home food availability) were associated with more healthy weight status. Odds ratios < 1 indicated the measure was associated with less healthy weight status.

^b ^P-values were calculated using ordinal regression; P-values < 0.05 are bolded.

## Discussion

This study is amongst the first to examine the associations of SET constructs from multiple domains (personal, social and physical environmental) with healthy weight status amongst mothers from socioeconomically disadvantaged neighbourhoods, a key risk group for overweight and obesity. Bivariably a number of personal, social and physical environmental variables predicted a healthy weight status, but multivariable results showed only a few key predictors that remained significant. Two of these factors related to women's commitment to making time for obesity-protective behaviours - healthy eating and physical activity - despite being busy with family commitments. These findings resonate with other research showing the difficulties, particularly related to lack of time and family commitments, faced by mothers when attempting to engage in health-promoting behaviours [11,22,32].

In addition to a personal commitment to making time for obesity-protective behaviours, the findings also showed that two familial factors remained predictive of healthy weight status in the multivariable model. Women whose families often ate healthy food with them were more likely to have a healthy weight. This could reflect a generally healthier eating pattern of the entire household, and/or the positive effects on women's eating of modelling or social observation of other family members eating healthily, which may protect against weight gain. While a number of past studies have examined the importance of family meal times for promoting dietary quality amongst children [33], we are not aware of other studies examining the impact of families eating healthily together on mothers' diets or weight. In contrast, frequency of familial encouragement of mothers' healthy eating was inversely associated with a healthy weight status amongst mothers. Acknowledging the inability to tease out cause and effect from this cross-sectional study, this finding could indicate that in families where the mother was already overweight or obese, family members more often encouraged the mother to eat healthily as a weight management strategy. It is noteworthy that eating healthy food with women represents a more active form of encouragement by family members compared with the relatively passive encouragement provided by verbally discouraging consumption of unhealthy food. As such, the present findings may indicate the utility of active family participation over passive encouragement for healthy eating in terms of producing or maintaining healthy weight status in mothers.

Collectively these findings attest to the significance of familial factors as correlates of mothers' capacities to engage in obesity-protective behaviours and of their weight status. Indeed the bivarable effect sizes (data not shown) of the associations between these factors and healthy weight status were found to be comparable to those between weight status and a variety of sociodemographic and behavioural characteristics examined in another READI sub-study (20). Furthermore, some familial factors (making time to eat healthy foods even when busy looking after family, and how often a mother's family eats healthy low-fat foods with them) actually demonstrated greater predictive ability than any of the sociodemographic or behavioural factors examined in that previous analysis. Such findings imply that intervention approaches to reduce the risk of obesity amongst mothers from disadvantaged neighbourhoods may benefit from taking a "whole family approach", in which other family members might be made aware of the importance of mothers taking time out for their own self-care relating to diet and physical activity, and encouraged to support this. Encouraging entire families to eat healthily together would also be important in this population, particularly in light of evidence that the practice of eating meals together as a family is reportedly less common amongst families experiencing socioeconomic disadvantage [34].

Strengths of this study include the focus on an underserved population at high risk of weight gain; the use of a theoretical basis to selecting correlates, which were also designed to capture factors most likely to be relevant to mothers; the large sample size that allowed control for confounding; and the large number of correlates assessed. Study weaknesses include the reliance on self-report data, and the cross-sectional study design. While the mothers in this study were in a healthy weight category at the time of the survey, without longitudinal data, or data on women's weight histories, we could not establish conclusively that there were 'obesity-resilient' over the longer term. We also cannot ascertain whether other correlates not examined in this study may have been important in predicting healthy weight (for instance, objectively-assessed features of women's local neighbourhoods reflecting their access to healthy foods or opportunities for physical activity).

## Conclusions

Acknowledging these weaknesses, this study provides novel insights into characteristics associated with a healthy weight status amongst mothers from socioeconomically disadvantaged neighbourhoods. Drawing on such characteristics could assist in developing intervention strategies to help other mothers in socioeconomically disadvantaged neighbourhoods to manage their weight. Such strategies might focus on planning for and prioritising time for healthy eating and physical activity behaviours, and including family members in and encouraging family mealtimes.

## Competing interests

The authors declare that they have no competing interests.

## Authors' contributions

AM carried out background research and drafted the manuscript. GA performed the statistical analyses and helped to draft the manuscript. KB and DC conceived the idea for and implemented the READI study, and developed the measures and methods. KB wrote the Discussion. DC helped interpret the findings and edited the draft manuscript. All authors read and approved the final manuscript.
